# Structural racism and the impact on incarcerated midlife women

**DOI:** 10.1186/s40695-022-00081-y

**Published:** 2022-11-05

**Authors:** Juana Hutchinson-Colas, Mary Cathryn Earnhardt, Afsara Mannan, James McGreevy, Gloria A Bachmann

**Affiliations:** 1 Women’s Health Institute, Rutgers Robert Wood Johnson Medical School, 125 Paterson Street, 08901 New Brunswick, NJ USA; 2 Rutgers Robert Wood Johnson Medical School, 125 Paterson Street, 08901 New Brunswick, NJ USA; 3 New Jersey Reentry Corporation, 591 Summit Avenue, 6 Floor, 07306 New Jersey, USA

**Keywords:** Structural racism, Incarcerated women, Midlife women

## Abstract

High recidivism rates indicate that current forms of imprisonment may be an ineffective response to problems that mainly burden those ensconced in poverty and marginalization. Homelessness, unemployment, racial disparities, drug use, and mental illness, disappear from public view when the afflicted individuals are relegated to a life behind bars. Women are the fastest growing prison population and most incarcerated women are from Black and Latinx groups. Structural racism encompasses the many ways in which society fosters racial discrimination through mutually reinforcing unfair systems of housing, education, employment, earnings, benefits, credit, media, health care, and criminal justice. In turn, this behavior reinforces discriminatory beliefs, values, and distribution of resources. Structural racism pervades every aspect of society, including the carceral system, from policing to prosecutorial decisions, pretrial release processes, sentencing, correctional discipline, and even reentry. Women constitute a minority within the carceral system, and as a result, their unique health care needs, especially during the midlife period, are inadequately addressed and often overlooked. There is also a general lack of gender sensitivity and special considerations in existing jail and prison policies and practices. This commentary highlights the impact of structural racism on the arrests and incarceration of women, and discusses their special health and wellness needs, with emphasis on midlife women. It also illuminates the need to address structural racism and its ripple effects within the carceral system.

## Background

Racism and discrimination are complex and generally deeply woven into the United States (US) society. These lead to significant social and economic injustices, which are frequently associated with repeated contact with the criminal legal system. Homelessness, unemployment, drug addiction, and mental illness, disappear from public view when impacted individuals a relegated to lives behind bars. Incarcerated women are typically from racially and economically disadvantaged backgrounds, that are characterized by violence and also physical and sexual abuse [1]. Many, having been subject to structural racism and poverty in their social and work communities, have these adverse social conditions, directly and indirectly, contributing to their incarceration. In addition to structural racism, the unique needs of incarcerated midlife women are highlighted. Policies governing jails and prisons in the United States (US) do not vary with gender, and thus, female and male prisons operate in the same manner; from the uniforms supplied that are made for male bodies, to the predominantly male guards in female quarters.

Incarcerated women are commonly affected by underlying mental health conditions, drug and alcohol problems, coercive relationships, financial difficulties, and debt [2, 3]. Adding to their burden, many incarcerated women also have dependent children and parents who are left in the same deprived environments [4]. Furthermore, women who are facing long sentences of more than 10 years or life sentences will eventually go through their midlife and postmenopausal years while incarcerated. Menopausal symptoms and emerging chronic conditions add to the immense discomfort and personal suffering that living in the adverse confines of a prison causes. Specifically, the distressing symptoms of menopause that many women experience, are not managed in the prison setting [5–7]. Data suggest that the overall provision of health care to incarcerated women falls short of what is defined by basic human rights [8]. To add to this dismal health care picture, there is a general lack of gender sensitivity and access to services outlined in existing prison policies and practices. The reality that incarcerated women have gender-specific needs, which are defined by their reproductive stage, is usually overlooked [1]. This translates to an overall negative impact on incarcerated women’s health, including the critical period of their moving from the reproductive years into the postmenopausal ones [1–3].

## Women in the criminal justice system

The ongoing segregation of minority groups, for example, Black individuals, in communities stricken by poverty, is associated with adverse birth outcomes, increased exposure to environmental toxins, decreased longevity, increased risk of chronic disease, and increased rates of homicide and other crimes [9]. Residential segregation in communities continues to systematically shape the quality of the neighborhood. Black people comprise a disproportionate share of those living in poverty-stricken neighborhoods and communities, where wide ranges of socio-economic vulnerabilities contribute to higher rates of crime, particularly violent crimes. The increased rates of violence and crimes in Black communities reinforce racialized police surveillance, arrest, conviction, and incarceration often for actions that are not sanctioned when taking place within non-Hispanic White communities [10]. Neighborhoods with enhanced policing also lack the resources to advocate for protection [5]. These circumstances contribute to the impact of structural racism seen in the practice of disproportionate and unjust sentencing in the criminal justice system of disadvantaged female minority communities [2]. Further, these inequities continue to reinforce discriminatory beliefs, values, and distribution of resources, which together affect the risk of both adverse exposure to the justice system and the risk of poor health outcomes while in prison [9]. This helps to explain the persistent racial disparities in prisons and the structural disadvantages that affect people of color long before they encounter the criminal justice system.

There were over 196,000 female incarcerations documented in the United States (US) between 1980 and 2019 [1]. This is due to several factors, including more expansive law enforcement efforts, sentencing laws, and post-conviction barriers to reentry, which uniquely affect women and incarceration rates [2]. Although there are fewer women in prisons than men, the rate of women imprisoned has been increasing twice as fast as that of men. By 2001, there were 27 per 100,000 White females aged 45 to 54 in the prison system, compared with 136 per 100,000 Black females [3]. In 2019, there were 194 per 100,000 women imprisoned in the US, with 75% from minority groups (83 per 100,000 Black and 63 per 100,000 Latinx) [2]. Of concern, the imprisonment rate for Black women was over 1.7 times the rate of imprisonment for White women, and Latinx women were imprisoned at 1.3 times the rate of White women [2]. The rate of incarceration among midlife women gradually increased between the years 1990–2010, with the highest rate among women between the ages of 50 and 54 [11]. Despite the lower incarceration rates in recent years, are still many midlife and older women behind bars because of several factors. These include harsh sentences for drug offenses, mandatory minimum sentences, and correctional practices that no longer use discretionary parole [11].

## Reasons for the incarceration of women

The United States incarcerates more women than any other nation in the world, because of the “war on drugs” [12]. Over 60% of the women in federal prisons are serving sentences for drug offenses and in many states, the rates are higher for alcohol and drug-related crimes [13]. However, the majority of offenses committed by women in prisons and jails are nonviolent and property crimes [14]. These offenses are often associated with conditions of poverty, intimate partner violence, and disadvantage. For example, drug laws are an important factor in the persistent racial and ethnic disparities observed in state prisons. Black individuals are nearly four times more likely as their White counterparts to be arrested for drug offenses and 2.5 times as likely to be arrested for drug possession [6]. This disparity exists despite the evidence that these two groups use drugs at roughly the same rate. From 1995 to 2005, Black people experienced 36% of drug arrests and represented 46% of those convicted for drug offenses, even though they only accounted for 13% of total drug users [7]. With later reform of the drug laws, and mandatory sentencing, Black people arrested on felony drug charges were still nearly twice as likely to receive a prison sentence compared to similarly situated White people [15]. In addition, an individual convicted of selling two ounces or possessing four ounces of heroin, morphine, opium, cocaine, or marijuana, is faced with a minimum of 15 years in prison.

It is important to note that convicted women are also victims. Their victim status is supported by a 2012 study that found that drug-involved offenders who experienced sexual and physical trauma in the year prior to enrollment in a drug court program were more likely to relapse within 18 months [16]. This study also found that if convicted, the individuals experienced depression because of the trauma, and the likelihood of relapse was even higher [16]. In addition, women prisoners are more likely to suffer from drug addiction than male prisoners [1]. Approximately 50% of women in US state prisons described themselves as daily users of drugs and 25% were under the influence of drugs at the time of their offense [17]. Additionally, over 30% said they had committed the crime, which brought them to prison, in order to obtain money to support their need for drugs [17].

Reasons for a woman’s conviction also include petty offenses and nonviolent crimes. Examples of petty offenses include loitering, indecent exposure, and possession of drugs. When crimes are committed by multiple individuals, many women endure the same punishment as their more significantly involved co-defendants, despite not directly participating in the crime [18]. Other reasons women are imprisoned include technical offenses such as probation and parole violations. These technical offenses can often lead to long-term imprisonment, which causes financial and mental distress for women [19]. In addition, there are associated moral costs, given that there may be no counsel available for those charged with misdemeanors, leading them to plead guilty due to the pressure of jail time. Thus, these women are then left with a criminal record that can be carried for a lifetime [20]. These low-level crimes are often prosecuted to extreme lengths rather than finding solutions that engage communities through policy changes. In addition, many women who are detained do not have the resources for bail or to hire a lawyer and in some instances are illiterate and unaware of their legal rights.

Several factors tied to structural racism often result in longer imprisonment than required. Racial bias against Black women in jury verdicts and sentencing also increases the length of sentencing. In addition, recent reforms of the sentencing laws resulted in decreased prison admittance in 2019 compared to 2018 but fewer early releases from prison. These circumstances result in many older incarcerated women who will continue to age behind bars because of the increased use of life without parole, mandatory sentences, and fewer early releases. These practices will continue to alter the overall prison age composition, and the prison system will be seeing a growing number of midlife and older women [3].

## Health care needs of midlife incarcerated women

As noted above, the number of midlife women in US jails and prisons is increasing, and little is known of their unique healthcare needs or of the extent to which these needs are met. Incarcerated women are more likely to have one or more chronic health conditions or disabilities than their community-dwelling counterparts [21]. These women have higher odds of having hypertension, asthma, arthritis, cancer, and a higher risk of infectious diseases such as hepatitis, HIV and tuberculosis. The emergence of the menopausal transition, with a range of physical and psychological symptoms, that can last over 10 years, can significantly affect the health needs of women in prison [3, 22]. Individuals experiencing the menopause transition while incarcerated report that menopause is “an important health concern” with disruptive and negative experiences resulting from the physical and psychological symptoms [23]. The menopausal symptoms can be distressing because of limited access to pharmacological and non-pharmacological symptom management. Furthermore, incarcerated women are not allowed commonly used lifestyle interventions to alleviate menopausal symptoms (e.g., layered clothing, cool drinks, frequent showers) [22]. Understanding the menopause experiences of women who are incarcerated is a critical step in identifying gaps in access to and quality of care for this underserved and growing population of older women in prisons and jails.

Medical providers caring for incarcerated females in their midlife and older years must utilize the framework of cultural competence and gender-responsive treatment that is free of racial bias. A gender-responsive framework is important because women are currently a relatively large segment of the prison and jail population in America [23]. This framework becomes important because women are likely to enter the correctional facility with chronic health conditions that require longer time for health care workers to evaluate and operationalize health care plans. It is also important to recognize the co-occurrence and effects of trauma, substance abuse, dysfunctional relationships, mental illness [24], and additional physiological changes of aging on women in custody.

Specific education and training of healthcare workers who care for incarcerated midlife women are necessary. In addition to benefiting the incarcerated patient, it will also help establish expectations amongst healthcare providers and potentially reduce any the stress of providing services for these women in need. Additionally, male correctional health care workers must be educated to display an appropriate supportive attitude towards older female patients. In addition to routine care, the benefits of nutrition, diet, and exercise are discussed to enhance the management of diabetes, hypertension, and osteoporosis. Overall, the prison environment must begin to address biases and meet standards that create an atmosphere that respects all incarcerated persons’ needs for health, safety and acceptance.

## Conclusion

Structural racism can explain the reason many women are in the web of incarceration and why many will continue to age behind bars reaching their midlife years and beyond. Alternatives to incarceration that preserve public safety and lead to improved health and public health outcomes ought to be rigorously pursued. Reforms to the judicial system that scale back the use of prison for low-level drug crimes should be commenced and resources directed to prevention and drug intervention programming. The conversation about structural racism must begin. This conversation will provide the catalyst to overcome barriers in the community that concentrate on poverty and crime. Effective preventative steps must be taken, such as greater access to health literacy programs, job training and the addition of local health clinics. Once incarcerated, women need to have access to a wide range of healthcare providers able to address the range of their healthcare needs. There are several opportunities to eradicate racism, sexism and discrimination in society in order to limit disparities and reduce incarceration rates while improving health care and rehabilitation of all women, including incarcerated midlife women. Legislation must continue to advocate, address and provide screening and health care for incarcerated aging women, who can still positively contribute to their families and society.

## Data Availability

not applicable.
